# Co-administration of iRGD with peptide HPRP-A1 to improve anticancer activity and membrane penetrability

**DOI:** 10.1038/s41598-018-20715-4

**Published:** 2018-02-02

**Authors:** Cuihua Hu, Xiaolong Chen, Yibing Huang, Yuxin Chen

**Affiliations:** 1 Key Laboratory for Molecular Enzymology and Engineering of the Ministry of Education, Jilin University, Changchun, 130021 China; 2 College of Life Sciences, Jilin University, Changchun, 130021 China

## Abstract

To improve the specificity and penetration of anticancer peptides against tumors, in this study, we examined the effects of co-administration of the membrane-active peptide HPRP-A1 and the tumor homing/penetrating peptide iRGD. iRGD peptide is widely recognized as an efficient cell membrane penetration peptide targeting to α_v_β_3_ integrins and neuropilin-1 (NRP-1) receptors, which show high expression in many tumor cells. The anticancer activity, cancer specificity and penetration activity *in vitro* and *in vivo* of the co-administered peptides were examined on 2D monolayer cells, 3D multi-cellular spheroids (MCS) and xenograft nude mice. Co-administration of iRGD and HPRP-A1 exhibited stronger anticancer activity and tumor specificity against A549 non-small cell lung cancer cells with NRP-1 receptor overexpression compared with HPRP-A1 alone. A549 cells showed uptake of the peptide combination and destruction of the integrity of the cell membrane, as well as adherence to the mitochondrial net, resulting in induction of apoptosis by a caspase-dependent pathway. The iRGD peptide dramatically increased the penetration depth of HPRP-A1 on A549 MCS and anticancer efficacy in an A549 xenograft mouse model. Our results suggest that the co-administration strategy of anticancer and penetrating peptides could be a potential therapeutic approach for cancer treatment in clinical practice.

## Introduction

During the past two decades, the development of cancer treatment has evolved from nonspecific cytotoxic agents to selective, mechanism-based therapeutics, such as chemotherapeutics, targeting agents, monoclonal antibodies and other targeted therapeutics. However, the efficacy of most anticancer drugs is limited due to the narrow therapeutic index, significant toxicity and frequently acquired resistance^1^. In particular, most drugs exhibit low activity against solid tumors because of the difficulty in entering tumor tissue and because the drugs only penetrate 3–5 cell diameters away from the blood vessels, which results in low efficacy and the development of drug resistance^2^. Thus, the development of strategies to improve targeting ability of anticancer drugs is greatly needed.

Cation anticancer peptides (ACPs) have been considered as novel therapeutic candidates due to their unique mechanism, broad-spectrum anticancer activity, low immunogenicity, and low tolerance^3^. The HPRP-A1 peptide, derived from the N-terminus of ribosomal protein L1 of *Helicobacter pylori*, has shown excellent antimicrobial activity and anticancer activity^4^. Our previous studies demonstrated the synergistic effect of HPRP-A1 with doxorubicin (DOX) and epirubicin (EPI) *in vitro* and *in vivo*^5^. In addition, a penetrating TAT peptide was chemically conjugated to HPRP-A1, and the resultant conjugated peptide exhibited an increased therapeutic index by about 152-fold compared with HPRP-A1^6^. Furthermore, we showed that HPRP-A1 functions by two mechanisms: by disrupting the cell membrane and inducing cell apoptosis^6^. Together these results suggest that HPRP-A1 is a promising candidate as an anticancer drug.

RGD was recognized as a targeting peptide that could bind selectively to α_v_β_3_ and α_v_β_5_ integrins screened by phage peptide libraries^7^. Internalizing-RGD, or iRGD, is a homing peptide with the sequence of CRGDKGPDC^8^. iRGD can increase vascular and tissue permeability when chemically conjugated with other drugs by specific binding with the NRP-1 receptor, which is overexpressed in tumor cells, and also allows co-administered drugs to penetrate into extravascular tumor tissue^9^. Teesalua *et al*. identified an important peptide sequence R/KXXR/K in which the C-terminal arginine is exposed by proteolytic cleavage, as the C-terminal exposure was the crucial factor for its biological activity, thus it was termed as C-end rule (CendR)^10^. The iRGD peptide contains a CendR motif, which is a specific sequence located in the C-terminal of peptides that require proteolytic processing for exposure of the biologically active motif required for binding to NRP-1, which triggers internalization of the peptide. iRGD homes to tumors through three steps: the RGD sequence binds to αv integrins on the tumor endothelium and then undergoes proteolytic cleavage, which results in the exposure of the binding motif for NRP-1^8^. The iRGD sequence, containing the RGD sequence, has physiological functions of RGD. Besides vascular attachment function through α_v_β_3_ and α_v_β_5_ affinition, iRGD could improve the cell and tissue penetration ability by conjugation or co-administration. Thus, iRGD is an idea tool for increasing the selectivity and penetrability of the peptides in our research. Although RGD is a simple, short and easily synthesized targeting peptide with only three amino acids, no report was published whether RGD peptide could increase the tumor cell selectivity by co-administration with other peptides. In this study, RGD was used as a control targeting peptide to compare with the effect of iRGD by co-administration therapy.

Neuropilins (NRPs) were first identified as receptors for class 3 semaphorins, a family of soluble molecules with neuronal guidance functions^11^. NRPs also play critical role in the development of both the nervous and vascular systems^12^. NRPs are co-receptors of many ligands, including semaphorins, vascular endothelial growth factor (VEGF), and many regulators of receptor tyrosine kinases (RTKs)^13^. In endothelial cells, NRP-1 interacts with VEGF receptor-2 (VEGFR2), enhancing proliferation, survival, migration and permeability. In addition, NRP-1 also binds the RTK c-Met, leading to increased proliferation, survival and migration of tumor cells in human glioma and pancreatic cancer^14^.

Previous studies have shown that iRGD improves the activity and specificity of several anticancer peptides, including ATAP^15^, m(KLA)^16^, TP5^17^, CDD^18^ and _D_(KLA)_2_^19^, by conjugating with these peptides. Kazuki *et al*. also observed that iRGD could enhance the tumor-specific delivery of different compounds (from a 0.6 kDa molecule up to a 130 nm particle) and suggested that the iRGD peptide may improve the performance range of several drugs, from cancer drugs to tumor imaging agents. Importantly, this enhancement effect did not require chemical conjugation between the drugs and the peptide^9^.

Traditional monolayer cell culture cannot reproduce some common characteristics of solid tumors *in vivo*, such as the production of the extracellular matrix^20^, which creates a major obstacle for examining drug penetration into tumor tissues. Sutherland *et al*.^21^ established 3D multi-cellular spheroids (MCS) in the 1970s. Large MCS (>200–300 *μ*m in diameter) contain three layers: proliferative cell populations at the periphery, an intermediate zone with viable and clonogenic but quiescent cells, and an inner necrotic core^22^. The MCS model has been demonstrated as a practical and simple model that reflects many properties of natural solid tumors. In addition, MCS is an ideal model to study drug penetration, along with multilayered cell cultures *in vivo*^2^.

In this study, we hypothesized that co-administration of HPRP-A1 with iRGD could help to improve the solid tumor penetration of the peptide. The mechanism of co-administration of HPRP-A1 and iRGD to improve the tumor penetration and specificity of anticancer peptide was investigated, as this would be important for the application of cationic ACPs as therapeutics. We used 3D MCS and A549 xenograft mouse model as models to evaluate the penetrability and mechanism of action of the peptide *in vitro* and *in vivo*, respectively.

## Results

### Peptide structure and anticancer activity *in vitro*

**Figure 1:**
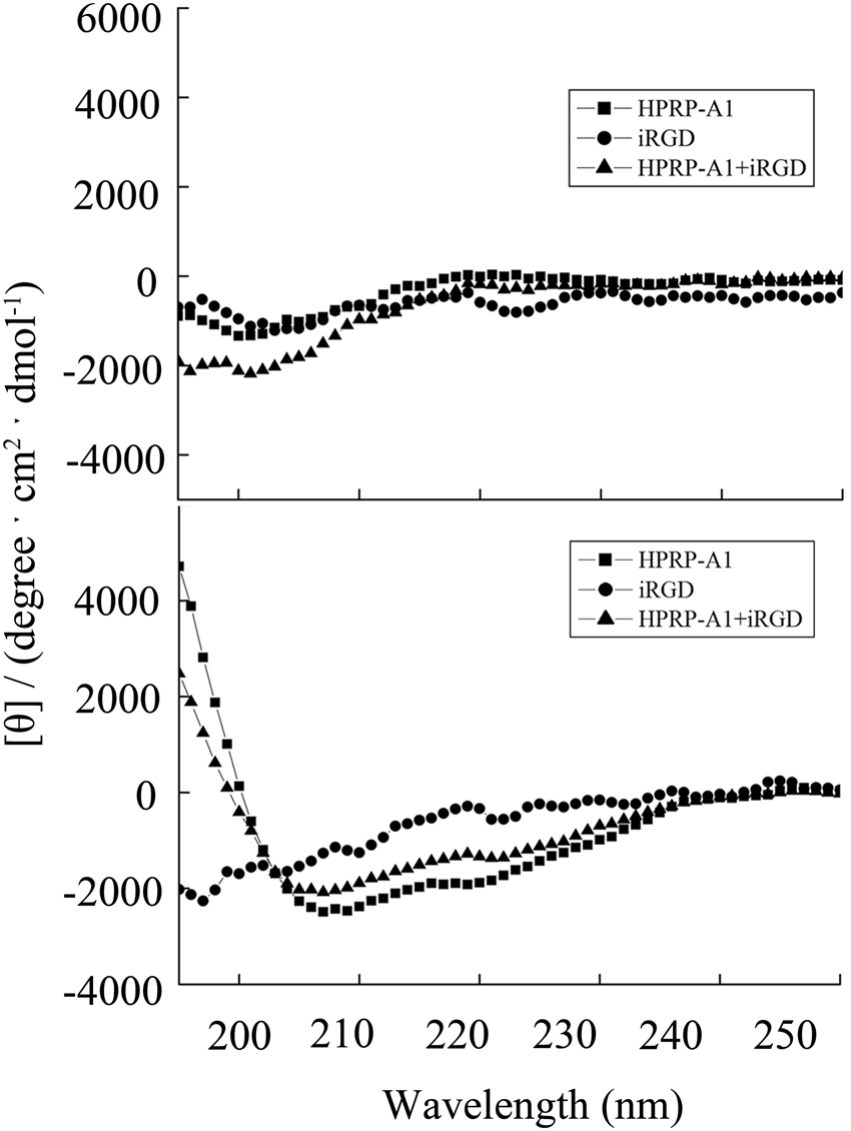
Circular dichroism spectra of peptides. Circular dichroism spectra of peptides (**A**) in benign medium (50 mM KH_2_PO_4_/K_2_HPO_4_ containing 100 mM KCl, pH 7.4) at 25 °C and (**B**) in the presence of 50% TFE at 25 °C.

We first examined the secondary structures of HPRP-A1 with or without co-administration of iRGD (concentration ratio HPRP-A1:iRGD = 1:1) using circular dichroism (CD) spectroscopy in benign condition as well as in an α-helix-inducing solvent in the presence of 50% trifluoroethyl alcohol (TFE), as described in Methods^23^. As shown in Fig. 1A, in benign condition, HPRP-A1 with or without iRGD co-administration and iRGD alone all exhibited a random coil structure. In contrast, in the presence of 50% TFE (Fig. 1B), iRGD exhibited a random coil structure, but HPRP-A1, both with or without iRGD, exhibited different degrees of an α-helical structure. However, the helical content of HPRP-A1 with iRGD was lower than HPRP-A1 peptide alone. The CD result of the peptides demonstrated that combination of iRGD could reduce the α-helical structure of HPRP-A1 peptide, suggesting a potential reducing toxicity against normal mammalian cells such as human erythrocytes^3^.

**Figure 2:**
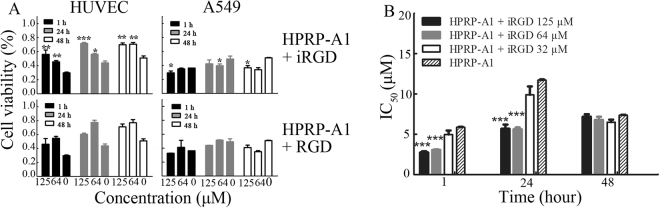
Cell viability and IC_50_ values in HUVECs and A549 cells treated with peptides. (**A**) Cell viability of HUVEC cells and A549 cells treated with HPRP-A1 alone or with iRGD or RGD was measured by MTT assay. Cells were cultured with 8 μM HPRP-A1 alone or in combination with iRGD (64 μM or 125 μM) or RGD (64 μM or 125 μM) for 1 h, 24 h or 48 h. (**B**) IC_50_ values of peptide HPRP-A1 with or without iRGD at the indicated concentration were measured by MTT assay at various times. *P < 0.05; **P < 0.01; and ***P < 0.001.

To study the anticancer activity and cancer cell selectivity of peptides, we used the A549 cell line, which exhibits overexpression of NRP-1^24^. Human umbilical vein endothelial cells (HUVEC) have low NRP-1 receptor expression^25^ and were selected as a control. We first measured the cell viability of both HUVEC and A549 cells treated with peptides using MTT assays (Fig. 2A). The RGD peptide was used as a comparison of iRGD. We treated cells with 8 μM of HPRP-A1 with or without 64 μM or 125 μM of iRGD or RGD for 1 h, 24 h and 48 h. Both 64 μM and 125 μM iRGD decreased the cytotoxicity of the HPRP-A1 peptide against the HUVEC cell line. However, in the A549 cell line, the cytotoxicity of HPRP-A1 peptide was increased after co-administration with iRGD. Notably, compared with iRGD, co-administration of RGD did not noticeably impact the effects of HPRP-A1 on both HUVEC and A549 cells.

We calculated the half maximal inhibitory concentration (IC_50_) values of HPRP-A1 and HPRP-A1 co-administered with 32 μM, 64 μM or 125 μM iRGD in A549 cells (Fig. 2B). The IC_50_ values of HPRP-A1 combined with 64 μM and 125 μM iRGD at 1 h and 24 h were approximately half of the IC_50_ values of HPRP-A1 alone. These data indicate that HPRP-A1 can induce rapid cancer cell death and that iRGD can enhance the killing activity of HPRP-A1 at a co ncentration above 64 μM. Thus, we explored the use of iRGD as a homing peptide for co-administration with HPRP-A1 in the following experiments.

### Apoptosis induction activity, cell cycle effect and caspase 3 activity of peptides

**Figure 3:**
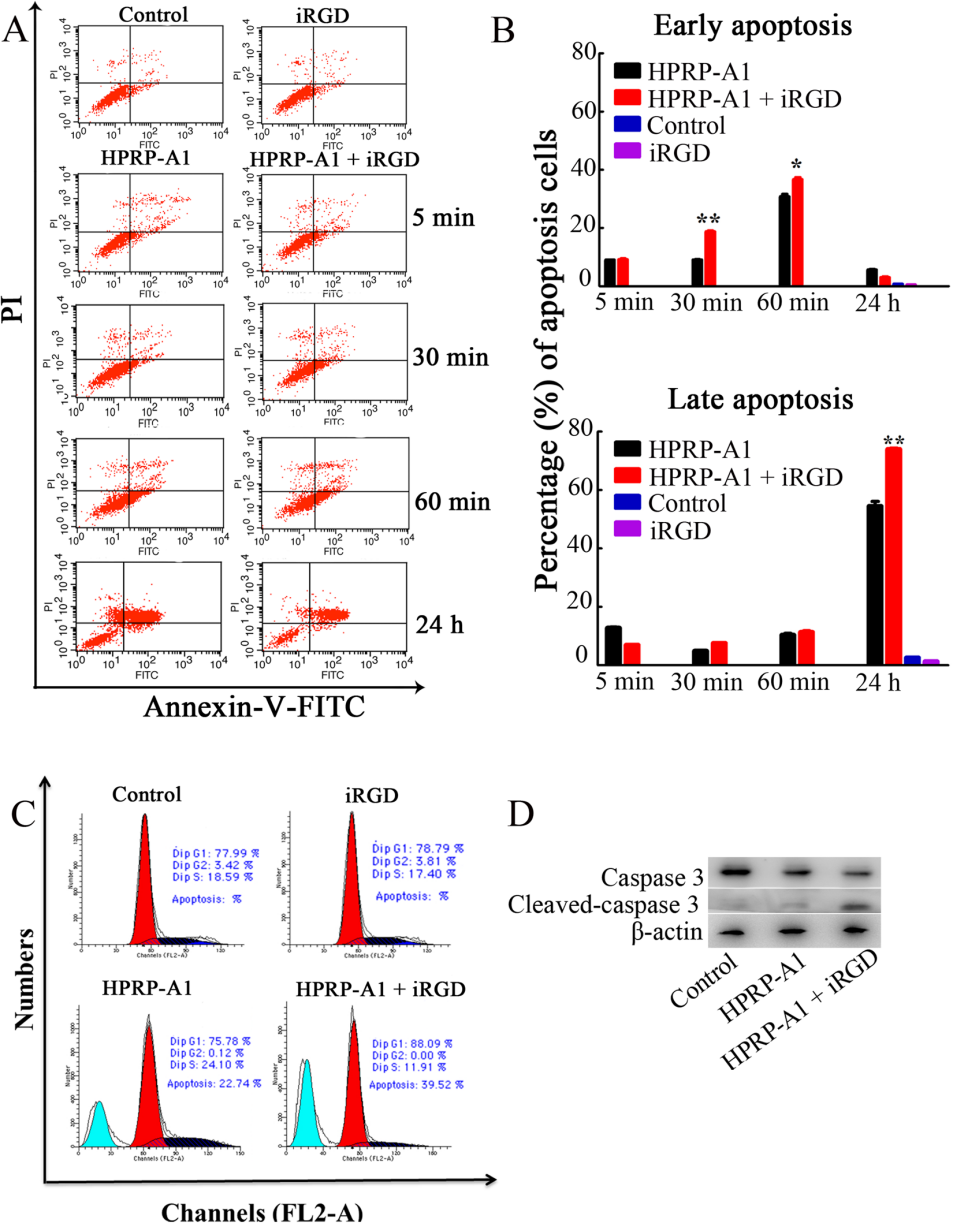
Apoptosis induction activity, cell cycle effect and caspase 3 activity of peptides in A549 cells. (**A**) A549 cells were cultured with 4 μM HPRP-A1 with or without 64 μM iRGD for 5 min, 30 min, 1 h or 24 h. The early and late apoptotic rates were measured by flow cytometry. (**B**) The percentage of cells in early and late apoptosis in A549 cells incubated with peptides as indicated in (**A**). (**C**) Cell cycle phase distribution in A549 cells after treatment with 4 μM HPRP-A1 with or without 64 μM iRGD for 24 h measured by flow cytometry. (**D**) The representative western blotting bands of Caspase 3 and Cleaved-caspase 3 of A549 Cells treated with 4 μM HPRP-A1 with or without 64 μM iRGD for 1 h. The bands of Caspase 3 and Cleaved-caspase 3 were cropped from one gel, as shown in Supplementary Fig. S2 with indication of molecular size. *P < 0.05; **P < 0.01; and ***P < 0.001.

A previous report showed that HPRP-A1 disrupted the integrity of the cell membrane and induced apoptosis in HeLa cells^3^. Thus, we examined the effects of HPRP-A1 on inducing early and late apoptosis in A549 cells using a FITC Annexin V/PI apoptosis detection kit. A549 cells were cultured with 4 μM HPRP-A1 with or without 64 μM iRGD for 5 min, 30 min, 1 h and 24 h, and the percentages of apoptotic cells were examined by flow cytometry (Fig. 3A,B). HPRP-A1 co-administered with 64 μM iRGD for 30 and 60 min induced early apoptosis in 18.68% ± 0.51% and 36.63% ± 0.95% of cells, respectively, while HPRP-A1 alone only induced early apoptosis in 9.05% ± 0.16% and 30.74% ± 1.20% of cells, respectively (Fig. 3A,B). After 24 h, only a few early apoptotic cells were detected and the number of late apoptotic cells increased dramatically. HPRP-A1 co-administered with 64 μM iRGD induced late apoptosis in 73.99% ± 0.55% of cells, while HPRP-A1 alone induced late apoptosis in about 54.43% + 2.34% of cells. These results demonstrate that co-administration with iRGD could significantly improve the apoptotic ability of HPRP-A1 in A549 cancer cells (*P* < 0.01).

We also examined the effect of HPRP-A1 with or without iRGD on the cell cycle by flow cytometry. A549 cells were cultured with 4 μM HPRP-A1 with or without 64 μM iRGD for 24 h (Fig. 3C). Consistent with the apoptosis results in Fig. 3A, HPRP-A1 co-administered with iRGD resulted in much higher numbers of cells in sub-G1 (40.33% ± 0.57%) than treatment with HPRP-A1 alone (22.03% ± 0.50%). Furthermore, HPRP-A1 co-administered with iRGD induced a G1 arrest (87.95% ± 0.19%) compared with HPRP-A1 alone or controls (78.39% ± 0.56% and 75.24% ± 0.76%, respectively).

To further verify the apoptotic activity of peptides, caspase-3 activity was examined by western blotting (Fig. 3D). In cells treated with HPRP-A1 together with iRGD, much higher levels of cleaved caspase 3 levels and lower levels of pro-caspase-3 were observed compared with both the controls and cells treated with HPRP-A1 alone. These results suggest that co-administration with iRGD promotes the ability of HPRP-A1 to induce apoptosis by a caspase-dependent pathway.

### Membrane destruction in A549 cells by peptides

**Figure 4:**
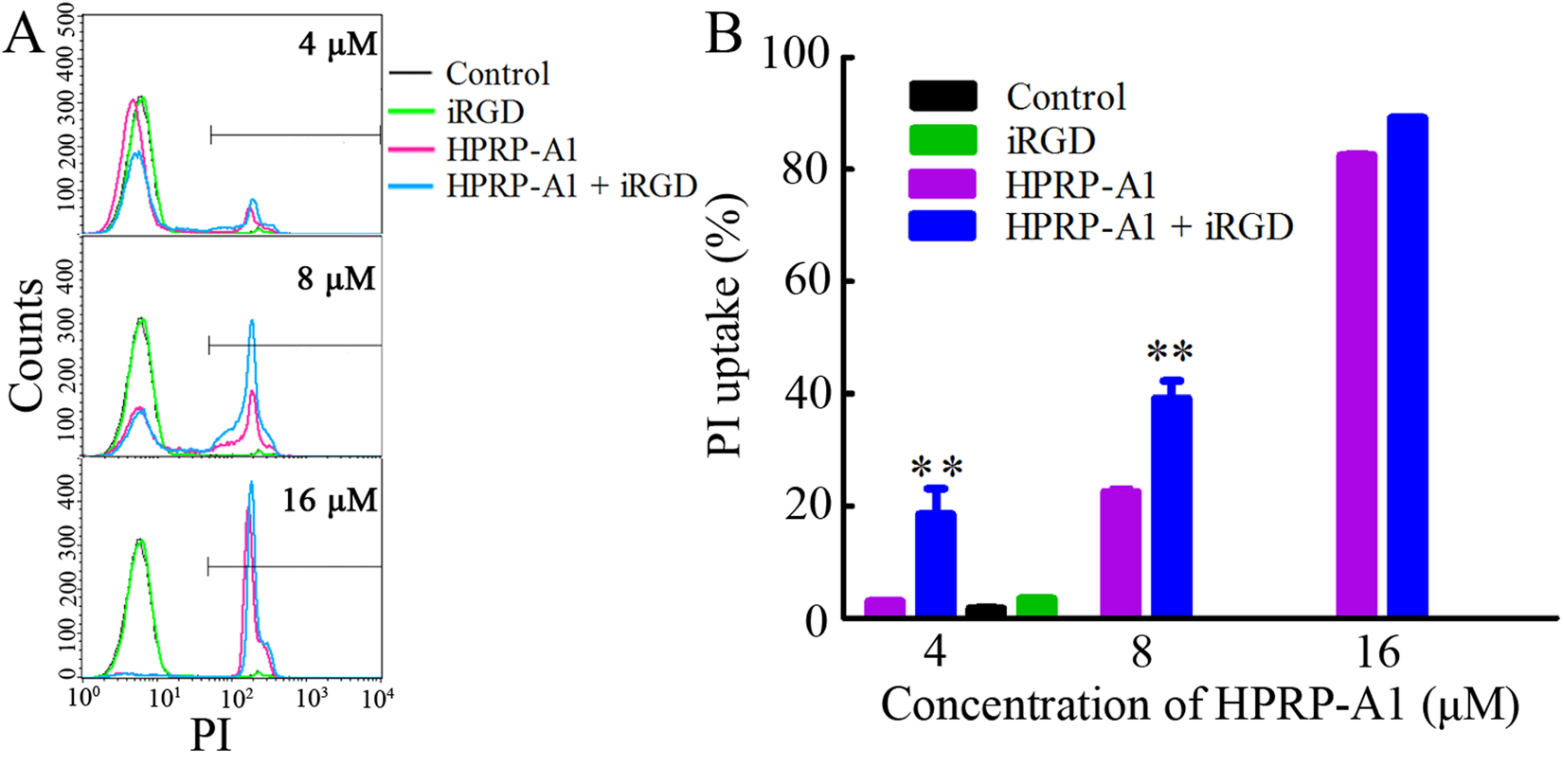
Membrane disruption in A549 cells by peptides. A549 cells were cultured with 4 μM, 8 μM or 16 μM HPRP-A1 with or without 64 μM iRGD for 1 h. Next, 5 μl PI was added and cells were incubated for 30 min. The fluorescence of PI was measured by flow cytometry. (**A**) Numbers of cells with PI uptake by flow cytometry. (**B**) The percentage of cells treated with various concentrations of HPRP-A1 and 64 μM iRGD peptide with PI uptake. *P < 0.05; **P < 0.01; and ***P < 0.001.

HPRP-A1 is a membrane-active peptide and iRGD is a targeted penetrating peptide^26,27^. Previous studies showed that membrane-active peptides can interact with the cell membrane, penetrate the phospholipid bilayer and eventually cause cell death^28^. Thus, we next examined the membrane disruption activity of HPRP-A1 with or without iRGD by flow cytometry. Propidium iodide (PI) was used as a probe to assess the membrane integrity because PI can enter the cell and combine with DNA when the integrity of the cell membrane is altered. We treated A549 cells with various concentrations of HPRP-A1 with or without 64 μM iRGD and cultured cells for 1 h. The cells were then stained with PI and examined by flow cytometry (Fig. 4A and B). The results showed that 4 μM HPRP-A1 with or without 64 μM iRGD could induce around 3.03% ± 0.15% and 18.55% ± 6.49% PI uptake, (*P* < 0.01). Treatment with cells with 8 μM HPRP-A1 induced around 22.56% ± 0.83% PI uptake, and co-administration with iRGD caused the PI uptake rate to increase to around 39.18% ± 5.31%. However, upon treatment with cells with 16 μM HPRP-A1, the PI uptake rate was almost 90% in both the HPRP-A1 alone group and HPRP-A1 and iRGD co-administration group.

### Cellular uptake of peptides

**Figure 5:**
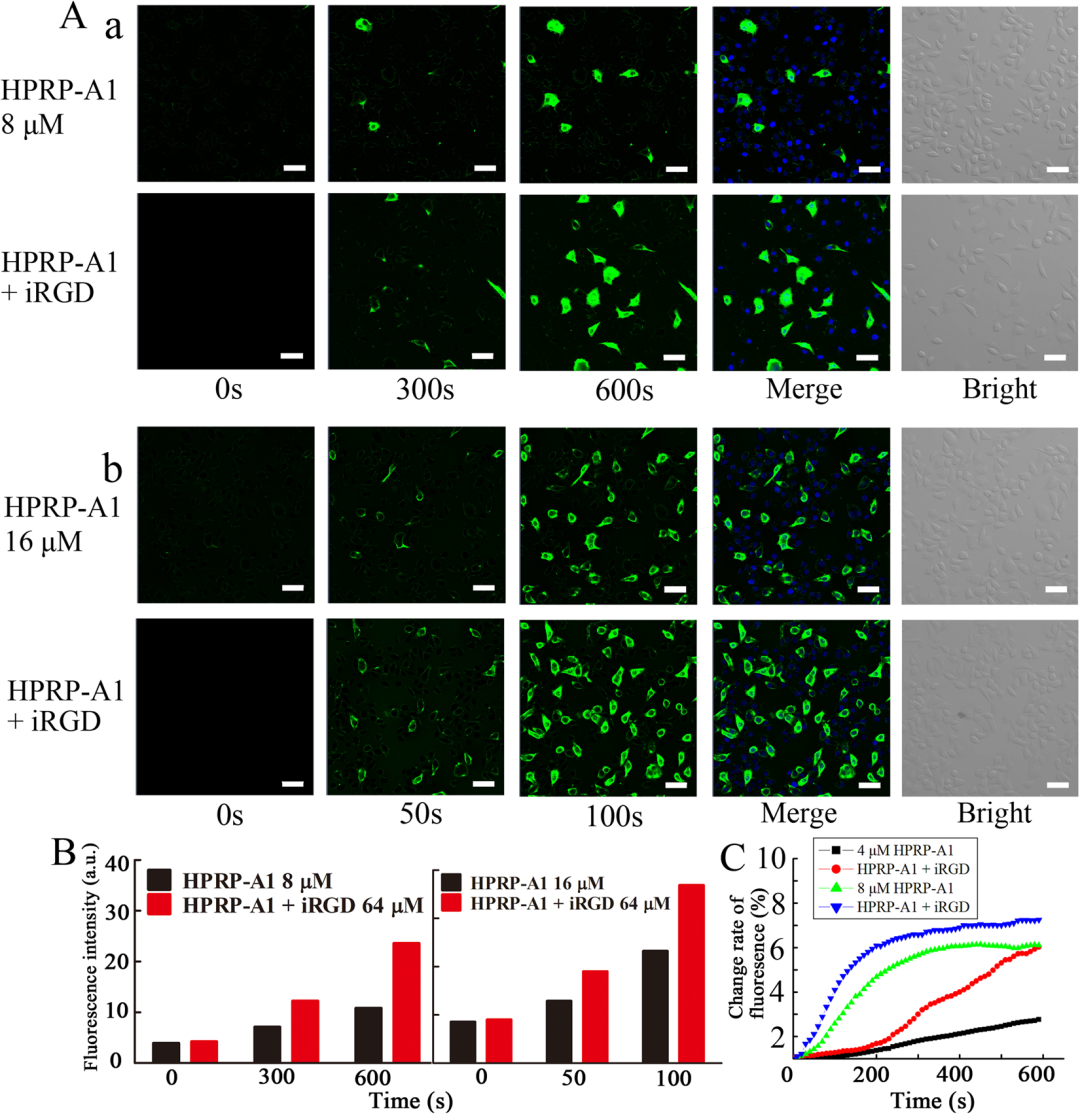
Cellular uptakes of peptides. (**A**) A549 cells were stained by Hoechst 33258. FITC-labeled HPRP-A1 with or without 64 μM iRGD was added to cells after two cycles, and the images were collected for 10 min at an interval of 10 s. The fluorescence was measured using a laser scanning confocal microscope. Blue color denotes nuclei, green color denotes FITC-modified peptide. (a) 8 μM FITC-HPRP-A1 co-administration with or without 64 μM iRGD. (b) 16 μM FITC-HPRP-A1 co-administration with or without 64 μM iRGD. (**B**) Fluorescence intensity of A549 cells treated with 8 μM or 16 μM FITC-labeled HPRP-A1 with or without 64 μM iRGD at each time point. (**C**) Fluorescence change rate of cells treated with FITC-labelled HPRP-A1 with or without 64 μM iRGD was recorded from 0 s to 600 s.

To explore the cellular uptake process of peptides, we performed laser scanning confocal microscope (LSCM) analysis (Fig. 5A). FITC-labeled HPRP-A1 at the concentration of 8 μM internalized into A549 cells from 300 s to 600 s, and the fluorescence intensity of cells with HPRP-A1 and iRGD co-administration was brighter than that of cells treated with HPRP-A1 alone. When the concentration of HPRP-A1 was increased to 16 μM, the fluorescence could be detected as early as 50 s and became very bright at 100 s. Figure 5B shows the quantitative values of fluorescence at each time point from Fig. 5A. The fluorescence intensity of the A549 cells, when treated with FITC-labelled HPRP-A1 peptide with co-administration of iRGD, was higher than the single peptide administration in 300 s, 600 s in 4 μM groups and 50 s, 100 s in 8 μM groups, revealing much more cellular uptake rates to the combination peptides compared with the single peptide. The fluorescence change rates of different groups are shown in Fig. 5C. The fluorescence change rates of cells treated with 4 μM FITC-labelled HPRP-A1 peptide showed a weak increase, while, that of cells treated with HPRP-A1 co-administration with iRGD increased sharply, and the final fluorescence potential was dramatically higher than the single HPRP-A1 group. The fluorescence change rate of cells treated with 8 μM FITC-labelled HPRP-A1 peptide with or without iRGD peptide exhibited similar increasing trend; in contrast, the increase rate and the final fluorescence intension in combination group was higher than the single peptide group. The result indicated that co-administration with iRGD could increase the rate and quantity of A549 cellular uptake. Together these results indicated that co-administration with iRGD significantly increased the uptake of HPRP-A1 in A549 cells. Supplementary video files show the cellular uptake process in more details (see Supplementary videos S1–S4).

### Mitochondrial depolarization, ROS generation and co-localization of peptides

**Figure 6:**
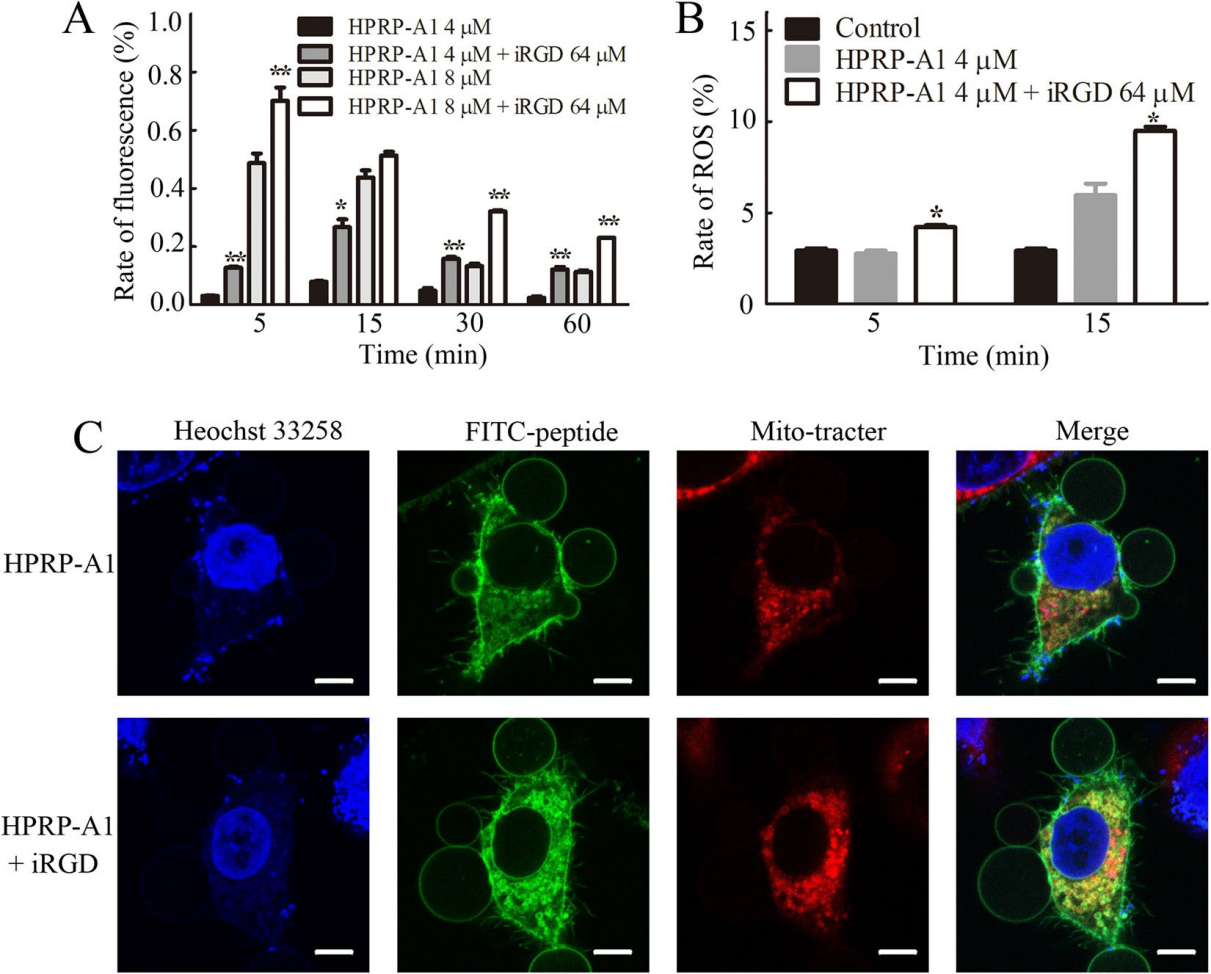
Mitochondrial depolarization, reactive oxygen species (ROS) generation and co-localization of HPRP-A1 with mitochondrial membrane. (**A**) Mitochondrial depolarization of cells. A549 cells were cultured with 4 μM or 8 μM HPRP-A1 with or without 64 μM iRGD for 5 min, 15 min, 30 min or 60 min. JC-1 probe was added according to the manufacturer’s instruction and the fluorescence was detected by flow cytometry. (**B**) ROS generation in cells. A549 cells were treated with 4 μM HPRP-A1 with or without 64 μM iRGD for 5 and 15 min, and ROS probe was added according to the manufacturer’s instructions. The fluorescence was detected using flow cytometry. (**C**) Co-localization of 8 μM FITC-labeled HPRP-A1 with or without 64 μM iRGD incubated with A549 cells for 30 min. Nuclei (blue color) and mitochondria (red color) were stained with Hoechst 33258 and Mito-Tracker^®^ Red, respectively, and cells were examined by laser scanning confocal microscopy. *P < 0.05; **P < 0.01; and ***P < 0.001.

We next measured the mitochondrial membrane potential by flow cytometry using the JC-1 probe. JC-1 is a fluorescent dye that shows red fluorescence under aggregation conditions in normal mitochondria, but shows green fluorescence when the mitochondrial membrane potential decreases. Thus, the change of mitochondrial membrane potential can be determined by the ratio of green fluorescence and red fluorescence using JC-1^29^. A549 cells were cultured with 4 μM or 8 μM HPRP-A1 with or without 64 μM iRGD for various time points, stained with JC-1 dye, and examined by flow cytometry (Fig. 6A). In the cells with HPRP-A1 co-administered with 64 μM iRGD, the mitochondrial membrane potential decreased much more than in cells treated with HPRP-A1 alone at both peptide concentrations (*P* < 0.01).

The increase of reactive oxygen species (ROS) generation can be one of the primary indicators of the disruption of the mitochondrial membrane^30^. We found that cells treated with HPRP-A1 co-administered with iRGD showed a higher level of ROS generation than cells treated with HPRP-A1 alone at 5 min and 15 min (Fig. 6B), which is consistent with the cell viability result showed in Fig. 2B.

The distribution of HPRP-A1 in cells by co-localization assays was investigated using LSCM (Fig. 6C). Interestingly, confocal images revealed that FITC-labelled HPRP-A1 and HPRP-A1 co-administration with iRGD could be taken up into A549 cells effectively and localized to mitochondria in a highly specific manner. In Fig. 6C, the merge images showed nearly complete overlapping between signals of FITC-labelled peptide and Mito-Tracker Green. The degree of overlapping was also demonstrated by Pearson’s correlation coefficient (Rr) using Image-Pro Plus software. The co-localization parameter was calculated using Rr, which describes the correlation of the intensity distribution between channels. The value of Rr ranges from −1.0 to 1.0, and 0 indicates no significant correlation and −1.0 indicates complete negative correlation^31^. The Rr vales in HPRP-A1-treated cells and cells treated with HPRP-A1 co-administered with iRGD were 0.620 and 0.778, respectively, which indicates that FITC-labeled HPRP-1 alone or co-administered with iRGD mainly co-localized at the mitochondrial membrane. Together these data revealed that HPRP-A1 alone or co-administered with iRGD localized to and disrupted the mitochondrial membrane to exert its anticancer activity.

### Penetrability of FITC-labeled peptide in A549 3D-MCS

**Figure 7:**
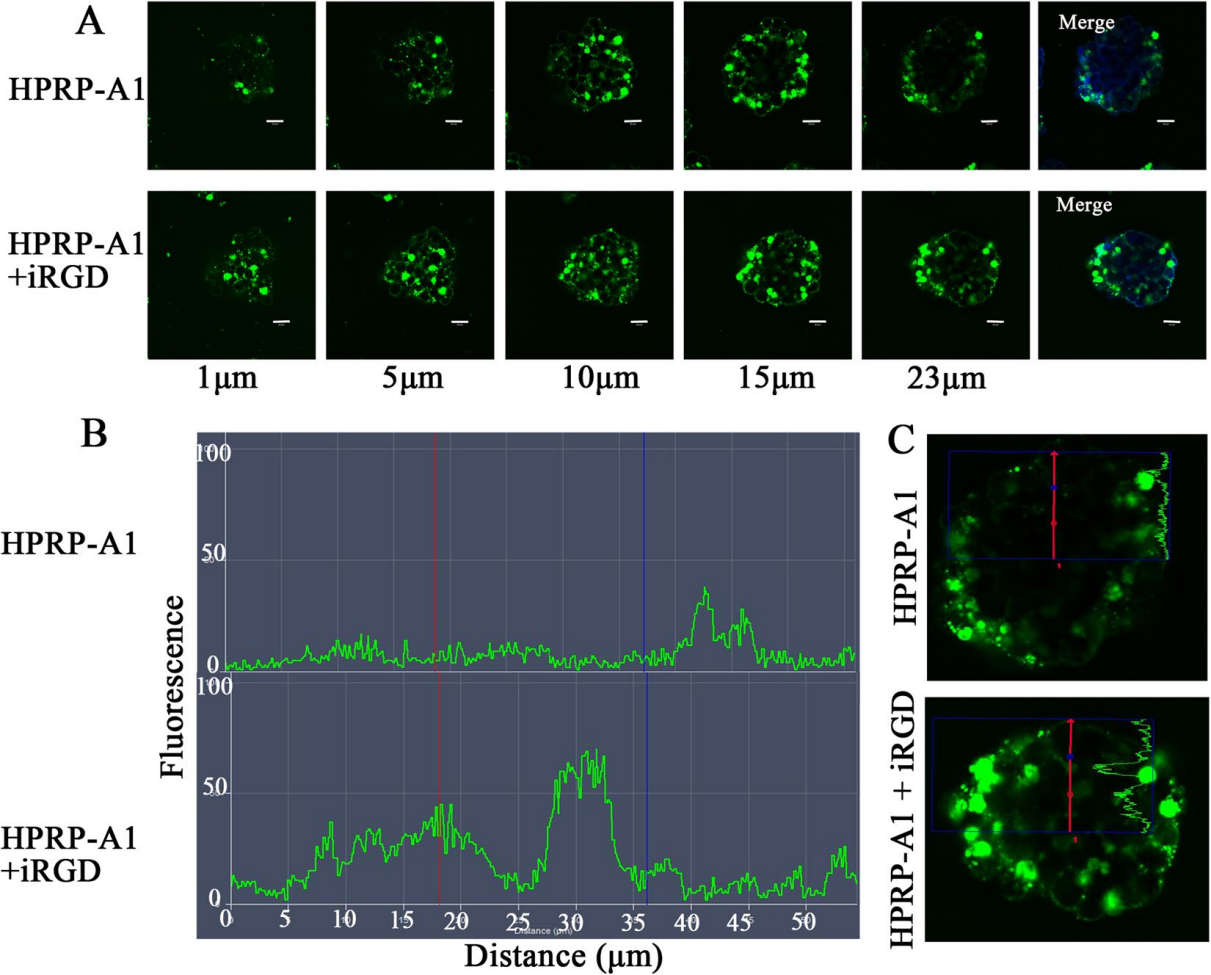
Penetrability of FITC-labelled peptide in A549 three-dimensional cellular spheres (3D-MCS). (**A**) Representative images of A549 MCS treated with 8 μM FITC-labelled HPRP-A1 with or without 64 μM iRGD for 30 min. (**B**) Changes of the fluorescence intensity of the 23^rd^ layer of MCS, from center to the margin. (**C**) Image of the 23^rd^ layer of MCS treated with HPRP-A1 alone (top) or treated with HPRP-A1 with iRGD (bottom). Green fluorescence indicates FITC-labeled HPRP-A1, the red arrows indicated the fluorescence change distance showed in Fig. 7B.

Previous studies showed that the homing peptide iRGD can enhance tumor-specific delivery of co-administered peptides^9^. We used an A549 3D cell model to further study the penetration ability of peptides. A549 3D MCS were cultured with 8 μM HPRP-A1 with or without 64 μM iRGD for 30 min, and the FITC-fluorescence was determined by LSCM using Z scan model (Fig. 7A). In the 15 μm layer of the MCS treated with HPRP-A1 alone, almost no green fluorescence was detected in the center of the MCS, indicating that HPRP-A1 may only penetrate into the 10^th^ layer of the cell sphere. However, in the MCS with HPRP-A1 with iRGD, more green fluorescence was detected even in the 23 μm layer, showing that co-administration with iRGD markedly improved the penetration ability of FITC-labeled HPRP-A1 into 3D MCS. Figure 7B shows the changes of fluorescence intensity of the 23^rd^ layer of MCS, from the center to the margin; the fluorescence intensity of the MCS with HPRP-A1 and iRGD was stronger than the MCS with HPRP-A1 alone. Figure 7C shows an image of the 23^rd^ layer of MCS. Together these results indicate that HPRP-A1 co-administered with iRGD significantly enhanced the penetration ability of HPRP-A1 peptide in A549 3D MCS.

### Effect of HPRP-A1 with iRGD on inhibiting tumor growth in nude xenograft mice

**Figure 8:**
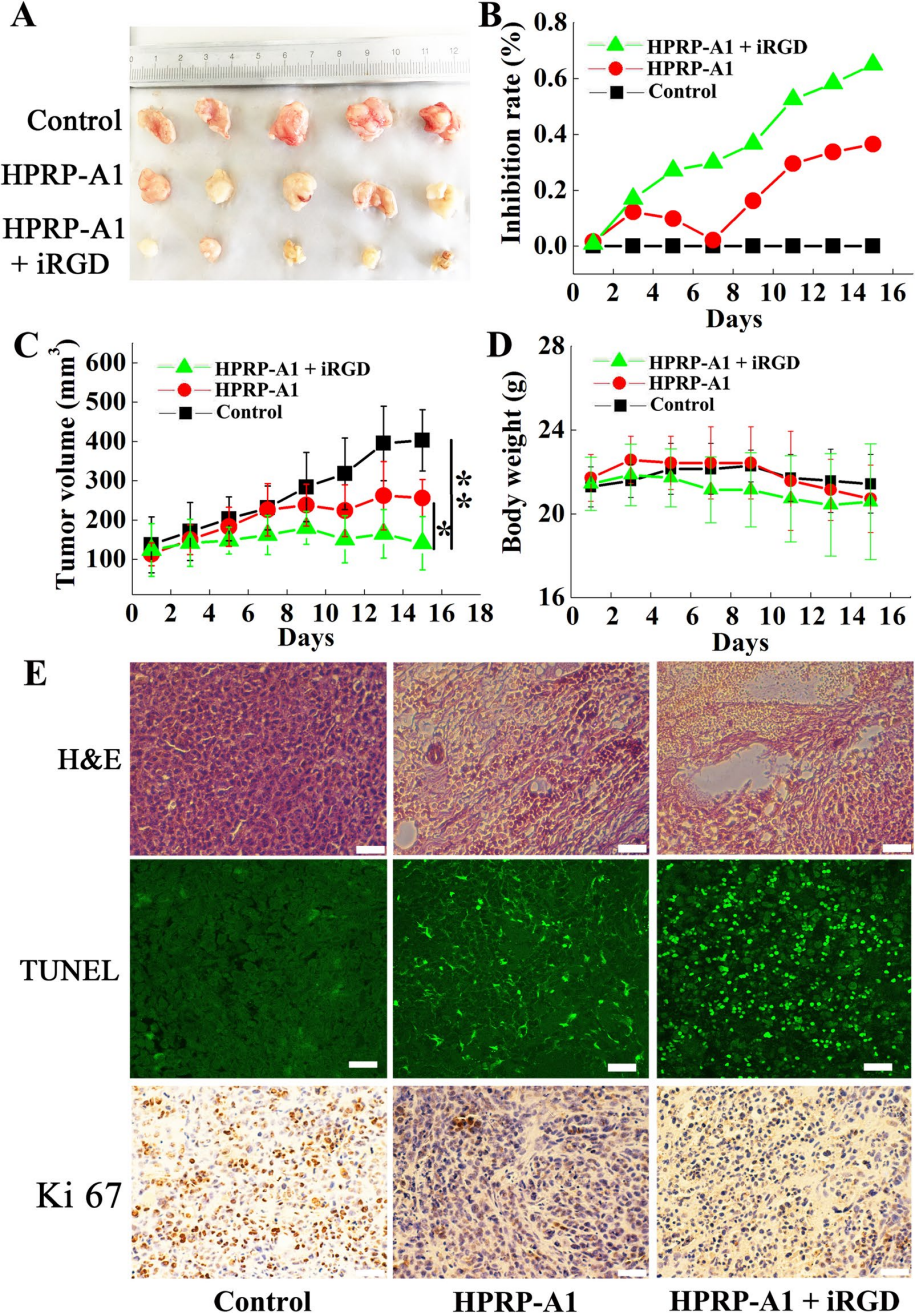
Effect of HPRP-A1 with or without iRGD peptide on inhibiting A549 cell growth in xenograft nude mice. (**A**) Representative images of tumors in all treatment groups. (**B**) Percentage inhibition of tumor growth in the treatment groups. (**C**) Tumor growth curves of each treatment group (n = 5 mice/group). (**D**) Body weight measured during the experimental period in each group. (**E**) Hematoxylin-eosin, TUNEL assays and Ki67 expression of the tumor tissues.

We next examined the effect of co-administration of HPRP-A1 and iRGD on the growth of tumors using nude xenograft mice *in vivo*. BALB/c nude mice were subcutaneously injected with A549 cells to create tumors. When tumors were about 120 mm^3^, the mice were randomly divided into three groups (n = 5 mice in each group). No statistically significant difference in tumor volume was detected among groups. HPRP-A1 peptide alone or with iRGD was directly injected into tumors once every 2 days. Saline solution was injected as the control. Tumor volume and body weight of mice in each group were recorded every 2 days. Both the tumor size and weight in the HPRP-A1 co-administered with iRGD group were dramatically smaller than those in the HPRP-A1 alone group and control group after intratumor injection for 16 days (Fig. 8A and Supplementary Fig. S1). In the control group, the tumor volume reached 400 mm^3^, while tumors were approximately 300 mm^3^ and 150 mm^3^ in the HPRP-A1 alone group and HPRP-A1 with iRGD group, respectively (Fig. 8C). These results showed statistical significance (*P* < 0.05, HPRP-A1 alone group compared with HPRP-A1 with iRGD group; *P* < 0.01, HPRP-A1 group compared with HPRP-A1 with control group). The inhibition rate in Fig. 8B clearly showed that mice treated with co-administration therapy experienced much greater tumor growth inhibition (65.0%) than animals treated with HPRP-A1 alone (36.47%). No difference in body weight among all groups was observed (Fig. 8D), indicating both the single HPRP-A1 and HPRP-A1 co-administration with iRGD did not reflect the body weight of the A549 xenograft mice.

We also performed hematoxylin-eosin staining (H&E), TUNEL assay and Ki 67 expression of the tumor tissue from all groups (Fig. 8E). The necrotic area in the tumor tissue from mice with HPRP-A1 co-administered with iRGD was much larger than that in mice with HPRP-A1 alone. TUNEL assays revealed much more apoptotic cells in the tissues from mice with HPRP-A1 co-administered with iRGD compared with tissues from mice with HPRP-A1 alone. Ki 67 protein expression in control group was much higher than that in HPRP-A1 alone or HPRP-A1 co-administration with iRGD. The expression content in HPRP-A1 peptide alone was higher than that in the combination peptide group, suggesting the proliferation inhibition of the combination peptides was stronger than that with HPRP-A1 peptide alone.

**Figure 9:**
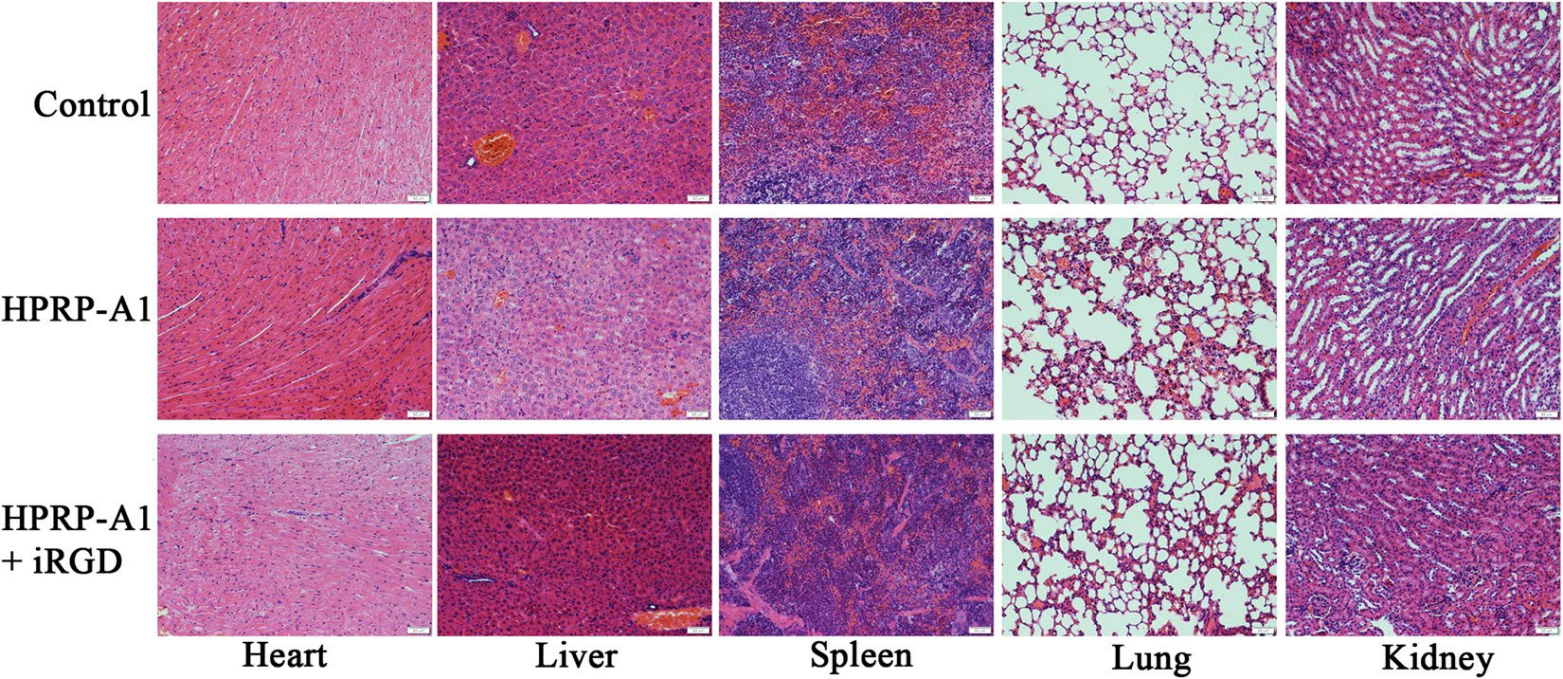
Hematoxylin-eosin staining of primary organs from the A549 cell xenograft mice model. Morphological details of indicated organs were investigated using hematoxylin-eosin staining. No significant pathological changes in heart, liver, kidney lung, and spleen were observed in nude mice following treatment with HPRP-A1 with or without iRGD.

The potential toxicity of HPRP-A1 with or without iRGD to the primary organs (heat, liver, spleen, lung and kidney) was determined by H&E assay at the end of the experiment. As shown in Fig. 9, there were no obvious changes in these organs after treatment with HPRP-A1 alone or together with iRGD.

## Discussion

Previous studies have demonstrated that a 15-mer cationic ACP HPRP-A1 induces apoptosis in HeLa cells and enhances growth inhibitory effects of DOX and EPI^3–5^. In this study, we demonstrated that the anticancer activity of HPRP-A1 can be improved when co-administered with iRGD *in vitro* and *in vivo* by disrupting the cell membrane and inducing fast apoptosis. The apoptosis induction occurs through the caspase pathway. Furthermore, the 3D MCS model showed that iRGD also enhances the selectivity of HPRP-A1 as well as the peptide penetration ability.

The HPRP-A1 peptide targets to the cytoplasmic membrane and exhibits a broad spectrum of antibacterial and antifungal activities as well as anticancer activity^4^, however, it possesses low specificity against tumor cells which is a common drawback of ACPs. In this study, the non-small cell lung cancer A549 cell line that overexpresses the NRP-1 receptor^24^ was used as the target cancer cell line, and HUVEC cells with low NRP-1 receptor expression^25^ were selected as a control. Our MTT results showed that the iRGD peptide increased the anticancer activity of HPRP-A1 in A549 cells, and decreased the toxicity of HPRP-A1 in HUVEC cells. Thus, co-administration of HPRP-A1 with iRGD resulted in improved selectivity to cancer cells compared with normal cells. The different NRP-1 protein expression may be attributed to the enhanced selectivity promoted by co-administration with iRGD.

As a membrane-active peptide, HPRP-A1 can induce rapid membrane disruption^6^. In the membrane disruption experiment (Fig. 4), co-administration of iRGD increased the PI uptake rate in A549 cells treated with 4 μM or 8 μM HPRP-A1 for 1 h. However, when A549 cells were cultured with 16 μM HPRP-A1, the PI uptake rates in cells treated with HPPR-A1 alone and cells treated with HPRP-1 and iRGD were similar, nearly 90%. This phenomenon may be attributed to the disruption of the entire cytoplasmic membrane at high concentrations of HPRP-A1, and therefore no difference in PI uptake rate could be observed. These results were also consistent with the cellular uptake assays using LSCM. We observed uptake of FITC-labeled HPRP-1 into cells within 100 s and 600 s at concentrations of 4 μM and 8 μM, respectively, and this uptake rate was enhanced by co-administration with iRGD.

After disrupting the cell membrane and entering cells, HPRP-A1 or HPRP-A1 combination with iRGD peptide was located in the mitochondrial membrane (Fig. 6C). In this study, the co-localization assay using LSCM demonstrated the exact location of the peptides in the cytoplasm. In our previous study, HPRP-A1 was shown to induce HeLa cell apoptosis by a caspase-dependent route, but there was no evidence that demonstrated an interaction between the peptide and the mitochondrial membrane. In this study, the co-localization assay provided morphological evidence for the reaction between the peptide and the mitochondrial membrane. As reported previously, cationic peptides are first attracted to the cytoplasmic membrane by the negatively-charged phospholipids; once electrostatically bound, their amphipathic property distorts the lipid matrix (with or without pore formation), resulting in the loss of membrane barrier function^32^. The eukaryotic mitochondrial membrane maintains a large transmembrane potential and has a high content of anionic phospholipids^33–35^. As demonstrated in the mitochondrial depolarization and ROS generation assays (Fig. 6A,B), HPRP-A1, as an amphipathic cationic ACP^5^, could attach to the mitochondrial membrane and neutralize the transmembrane potential at low concentrations, and cellular apoptosis consequently occurs. This result reveals that the mitochondrial membrane could be the secondary target of the HPRP-A1 peptide in addition to the cytoplasmic membrane as the primary target^36^. At high concentrations, HPRP-A1 would cause the thorough disruption of membranes through a necrotic path^37^.

In this study, we showed that HPRP-A1 could induce fast apoptosis in A549 cells by disrupting the mitochondrial membrane, and the apoptosis induction could be enhanced by co-administration with iRGD. The fast apoptosis induction was observed as soon as 30 min after culturing the peptide with A549 cells. The changes of electric potential of mitochondria and ROS release^38^ were also detected at 5 min and 15 min, respectively (Fig. 6A,B). Furthermore, the increase of caspase 3 level demonstrated the apoptosis occurrence occurred via the mitochondrial pathway (Fig. 3D).

To investigate the penetration ability of HPRP-A1 after co-administrating with iRGD, the 3D-MCS model was established to mimic the avascular tumors *in vitro*. As Fig. 7 showed, co-administration with iRGD increased the penetration depth of HPRP-A1 on A549 3D-MCSs. The possible mechanism of the co-administration peptides-induced penetration should be resulted from NPR-1 expressed on A549 cell membrane. As Sugahara *et al*. mentioned, iRGD is a cyclic peptide (CRGDK/RGPDC) which initially binds to α_v_-integrins using its vascular recognition (CendR) motif, where it gets proteolytically cleaved and the truncated peptide (CRGDK/R) gains affinity for neuropilin-1 (NRP1)^10^. When iRGD binds to NRP-1, a cell internalization and trans-tissue transport pathway is activated^39^, importantly, this effect did not require the drugs to be chemically conjugated to the peptide^9,10^.

Based on the *in vitro* anticancer activity research, we further tested the *in vivo* activity of HPRP-A1 co-administration with or without iRGD on A549 xenograft nude mice. As shown in Fig. 8 and Fig. S1, the tumor volume and tumor weight were all inhibited significantly by administration of the mixed peptides, the proliferation inhibition effect was also verified by the H&E test and Ki 67 expression assay on tumor slices. The *in vivo* test results were consistent with the *in vitro* anticancer activity, that is, co-administration of HPRP-A1 and iRGD exhibited stronger anticancer activity in both *in vitro* and *in vivo* experiments. The mechanism of the *in vivo* activity was detected using TUNEL, the results demonstrated that an apoptosis induction of the A549 xenograft was occurred by the administration with HPRP-A1 and iRGD, which was consistent with the apoptosis induction activity of the mixed peptides *in vitro* showed in Fig. 3A,B; Meanwhile, the body weight data (Fig. 8D) and H&E test (Fig. 9) indicated that HPRP-A1 co-administration with or without iRGD showed no toxicity against major organs of the A549 xenograft nude mice.

## Material and Methods

### Material

The FITC Annexin V/PI apoptosis detection kit was purchased from BD Biosciences (USA). The Hoechst 33258 dye detection kit, PI dye detection kit, JC-1 mitochondrial membrane potential detection kit and ROS assay kit were provided by Solarbio Life Science (Beijing, China). MitoTracker^®^ probes, and the Pierce™ BCA protein assay kit were provided by Thermo Fisher Scientific (MA, USA). FITC-HPRP-A1 were provided by GL Biochem Ltd. (Shanghai, China). The one step TUNEL apoptosis assay kit and RIPA lysis buffer were provided by Beyotime Company (Beijing, China). Thallium (III) trifluoroacetate was purchased from Tokyo Chemical Industry (Japan). The human lung cancer cell line A549 and human umbilical vein endothelial cells (HUVECs) were obtained from the Institute of Biochemistry and Cell Biology, Shanghai Institute for Biological Sciences, Chinese Academy of Sciences (Shanghai, China).

### Peptide synthesis and purification

The anticancer peptide HPRP-A1 (Ac-FKKLKKLFSKLWNWK-amide), homing peptide iRGD (Ac-CRGDKGPDC-amide) and RGD (Ac-RGD-amide) peptides were synthesized using solid-phase methods on MBHA rink amide resin by 9-fluorenyl-methoxycarbonyl chemistry as described previously^40^ and were purified by reverse-phase high-performance liquid chromatography. iRGD is a disulfide-based cyclic peptide; this reaction was oxidized by thallium trifluoroacetate on solid-phase resin after the linear chain amino acid completed as described previously^41^. The purified peptides were further characterized by mass spectrometry and amino acid analysis.

### Circular dichroism spectroscopy of peptides

The helical structures of peptides were detected by CD spectroscopy using a Jasco J-810 spectropolarimeter (Jasco, Tokyo, Japan) at 25 °C under KP buffer (50 mM KH_2_PO_4_/K_2_HPO_4_, 100 mM KCl, pH 7) as well as in an α-helix-inducing solvent, TFE buffer (50 mM KH_2_PO4/K_2_HPO_4_, 100 mM KCl, pH 7 buffer, 50% TFE)^23^. The values of molar ellipticities of the peptide HPRP-A1 with or without iRGD at a wavelength of 208 nm and 222 nm were used to estimate the relative helicity of the peptides.

### Cell culture

A549 and HUVEC cell lines were cultured in Dulbecco’s Modified Eagle medium (DMEM) (Gibco Life Technologies, Thermo Fisher Scientific, MA, USA) with fetal bovine serum (FBS) (10% v/v), penicillin (100 U/ml), and streptomycin (100 U/ml) in a humid atmosphere at 37 °C with 5% CO_2_.

### Cell viability assay

A549 cells (1 × 10^4^) were plated in triplicate in a 96-well microtiter plate. After 12 h, the medium was replaced with 100 μl of fresh medium containing various concentrations of peptides or medium without peptide as a negative control. After 1 h, 24 h or 48 h, 20 μl of MTT was added and cells were cultured at 37 °C for 4 h. Dimethyl sulfoxide was added to dissolve the formazan crystals and the absorbance at 492 nm was measured with a microplate reader (GF-M3000; Gaomi Caihong Analytical Instruments Co., Ltd. Shandong, China). The IC_50_ and cell viability were calculated. The MTT assays were repeated in triplicate.

### Flow cytometric analysis

A549 cells in the logarithmic growth phase were seeded (2–3 × 10^5^ cells/well) in 6-well plates. After cells were cultured in 37 °C, 5% CO_2_ for 24 h, the culture medium was replaced with fresh medium containing HPRP-A1 peptide with or without iRGD. After incubation for various time points, the cells were washed with cold PBS three times and digested with trypsin for 1 min. DMEM with 10% FBS was added to stop trypsinization, and the cells were collected and centrifuged under 0.2 (rcf) g three times. The cell pellet was washed three times with cold PBS and then stained using the PI kit according to the manufacturer’s instructions for membrane destruction test. The cell precipitate was re-suspension and fixation in 95% ethyl alcohol for 1 h at −20 °C, then stained by PI kid according to the manufacturer’s instructions. As for apoptosis analysis, after re-suspension, the cells were stained by Annexin-V-FITC/PI apoptotic detection kit according to the manufacturer’s instructions. The fluorescence was measured using flow cytometry (FACSCalibur, Becton-Dickinson, San Jose, CA, USA).

### Western blotting

A549 cells treated with HPRP-A1 with or without iRGD were washed with ice-cold PBS and solubilized in RIPA lysis buffer. After incubating on ice for 2 h, the insoluble materials were removed by centrifugation at 12000 rpm for 15 min at 4 °C. Accurate protein concentrations were determined using the bicinchoninic acid (BCA) protein assay kit. Protein samples (80 μg) were separated on 12% SDS-PAGE gels and then transferred to polyvinylidene fluoride membranes. Membranes were blocked for 2.5 h with 5% skim milk in PBS at room temperature and blotted with the primary antibody against caspase-3 (1:5000 dilution; Cell Signaling Technology, MA, US 9665)^42^ overnight at 4 °C. β-actin antibody (Santa Cruz Biotechnology, CA, US. sc-4778) was used at 1:1,000 dilution as a loading control. After washing by PBS with Tween-20, the membranes were incubated with horseradish peroxidase-conjugated secondary antibodies (abcam, MA, US) for 2 h at room temperature. The blots were detected using an enhanced chemiluminescence kit (Millipore, USA). The results were visualized on the Tanon-5200 Chemiluminescent Imaging System (Tanon Science & Technology Co., Ltd., Beijing, China)^43^.

### Cellular uptake and co-localization of the peptides

A549 cells were cultured in glass-bottomed dishes for 24 h. For peptide cellular uptake studies, after 24 h of culture with DMEM containing 10% FBS, cells were labeled with Hoechst 33258 for 30 min at 37 °C. Cells were washed three times with PBS, and 100 μl DMEM culture medium was added to the dish. Cells were then scanned by LSCM (LSM710, CarlZeiss, Oberkochen, Germany) using a time series model, at 10 s per cycle, with a total of 60 cycles. After two cycles, 900 μl of medium containing HPRP-A1 with or without 64 μM iRGD were added using a microsyringe and 60 LSCM images were obtained. The LSCM images were processed and analyzed using the ZEN lite 2012 image software, CarlZeiss, Oberkochen, Germany. The fluorescence change rate was calculated by the following equation: fluorescence change rate = (fluorescence value at the experimental time point/fluorescence value at time 0) × 100%.

For the co-localization tests, the cells were treated with 8 μM FITC-labeled HPRP-A1 with or without 64 μM iRGD and incubated for another 30 min. Cells were washed twice with ice-cold PBS and stained with 200 nM MitoTracker^®^ Red FM to label mitochondria or Hoechst 33258 to label nuclei according to the manufacturer’s instructions. The stained cells were imaged using a LSCM. Composite images were created by overlapping the images obtained from individual channels. The co-localization parameter was calculated using Image-pro plus 6.0.

### Mitochondrial depolarization assay

The mitochondrial membrane potential was measured using the fluorescent dye JC-1 (Molecular Probes, Beijing, China)^14^. After treatment with 4 μM or 8 μM HPRP-A1 with or without 64 μM iRGD for various times (5, 15, 30 or 60 min), A549 cells were incubated with 5 μg/mL JC-1 for 30 min. The peptide-treated cells were then collected by centrifugation and analyzed using flow cytometry. The ratio of red to green signal was calculated.

### ROS assay

A549 cells were cultured in a 6-well microtiter plate. After 24 h, 10 μM of the ROS probe DCFH-DA was added to cells and cells were cultured at 37 °C for 20 min. Cells were washed with fresh culture medium twice and then treated with 4 μM HPRP-A1 with or without 64 μM iRGD for 5 or 15 min. The cells were then harvested by centrifugation, washed twice with PBS, and suspended in PBS. The intracellular ROS levels were then immediately examined by measuring the fluorescence intensity of DCF using flow cytometry.

### Penetrability assay of peptides using 3D cell spheres

To prepare the A549 MCS, Matrigel (BD Bioscience) was coated on the bottom of a 24-well plate at 4 °C and the plate was incubated at 37 °C. A549 monolayer cells were cultured in the 24-well plate to give a single-cell suspension at 37 °C in a 5% CO_2_ humidified atmosphere. A549 cells formed 3D spheres in approximately 7 days^44^. To evaluate the penetrability of peptides, the FITC-labeled peptide was cultured with A549 MCS for 30 min, and the MCS was scanned using LSCM (LSM710, CarlZeiss) by horizontal model from the top to the middle of the sphere, once at every 1 μm. The fluorescence curves were produced using ZEN lite 2012 image software.

### Animals and antitumor efficacy in xenograft mice

Six to eight week-old female BALB/c nude mice, weighting from 18–22 g purchased from Beijing Weitonglihua Co., Ltd. China, were housed in a Good Laboratory Practice laboratory. A549 cells (1 × 10^7^) were subcutaneously injected into the right posterior limb of mice. When the tumors grew to 150 mm^3^, the xenograft mice were randomly divided into three groups (n = 5 per group). Based on the *in vitro* results and the previous study^5^, 10 mg/kg of HPRP-A1 with or without 30 mg/kg of iRGD were co-administrated directly into A549 tumors in the xenograft mouse model. Tumors were directly injected with saline (control), 10 mg/kg HPRP-A1 alone, or 10 mg/kg HPRP-A1 with 30 mg/kg iRGD every 2 days, totally for 16 days^5^. The tumor size was calculated using the following formula: (length × width^2^)/2. The inhibition rate of tumor growth (%) was calculated using the following formula: (tumor volume in control group − tumor volume in test group)/tumor volume in control group ×100%^5^. Mice were sacrificed at the end of the experiment. Tumor tissues and main organs (heart, liver, spleen, lung and kidney) were dissected for histology observation. The organs were fixed in 10% paraformaldehyde, and tissues were embedded in paraffin, sectioned at a thickness of 5 μm, and stained by H&E TUNEL assay using the one step TUNEL apoptosis assay kit^5,45,46^, and cultured by Ki 67 antibody (Wanleibio, Shenyang, China). All care and handling of animals were performed in accordance with the guidelines of the animal ethics committee of Jilin University (Approval No. JLUSWLL003, Jilin, China).

### Statistical analysis

Data are expressed as means ± standard error (SE) of the mean. The data were analyzed through one-way ANOVAs followed by post hoc Duncan test (SPSS 17.0). *P* < 0.05 was considered significant.

### Data availability

All data generated or analyzed during this study are included in this published article (and its Supplementary Information files).

## Conclusions

In conclusion, in this study a homing peptide iRGD was used for co-administration with a membrane-active anticancer peptide HPRP-A1 to increase its anticancer activity. Cell viability analyses indicated that iRGD could improve the selectivity and anticancer activity of HPRP-A1 in cancer cell lines. iRGD enhanced the membrane disruption activity and cellular uptake of HPRP-A1, resulting in an increase of the percentage of apoptotic cells and ROS generation, as well as the mitochondrial depolarization of A549 cells. Furthermore, iRGD could dramatically increase the penetration depth of HPRP-A1 in the A549 3D MCS model. *In vivo* analyses also showed that HPRP-A1 co-administered with iRGD inhibited solid tumor growth and exhibited better anticancer activity compared with HPRP-A1 alone. iRGD promoted cell apoptosis through the mitochondria-dependent pathway induced by HPRP-A1 both *in vitro* and *in vivo*. We believe that the co-administration of HPRP-A1 and iRGD could be a promising approach as an anticancer therapeutic strategy in clinical practice.

## Electronic supplementary material

Supplementary information

Video S1

Video S2

Video S3

Video S4
