# Supplementary material for: Co-administration of iRGD with peptide HPRP-A1 to improve anticancer activity and membrane penetrability
Source: Sci Rep. 2018 Feb 2;8:2274. doi: 10.1038/s41598-018-20715-4 (PMC5797073; doi:10.1038/s41598-018-20715-4)
Supplement: Supplementary file 1 — Supplementary information [file 41598_2018_20715_MOESM1_ESM.pdf]

Co-administration of iRGD with peptide HPRP-A1 to improve anticancer activity and membrane penetrability

Cuihua Hu[1,2], Xiaolong Chen[1,2], Yibing Huang[1,2], Yuxin Chen[1,2*]

Supplementary Materials:

Figures S1-S2

Video S1-S4

Supplementary Figure S1-S2

Figure S1

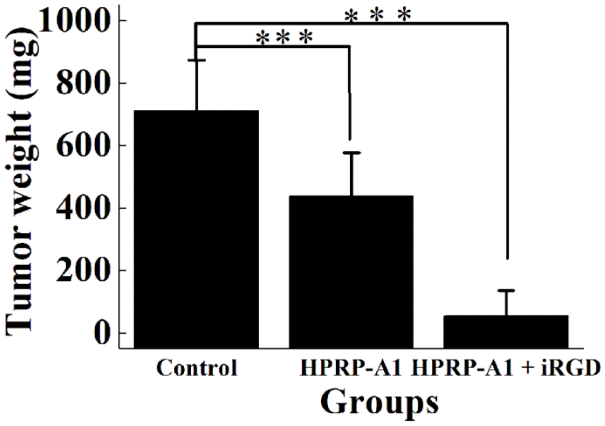

Figure S1: Average tumor weight in each treatment group at the termination of study. The average tumor weight in each treatment group was weighted at the termination of the study. $^{*}P < 0.05$; $^{**}P < 0.01$; and $^{***}P < 0.001$.

Figure S2

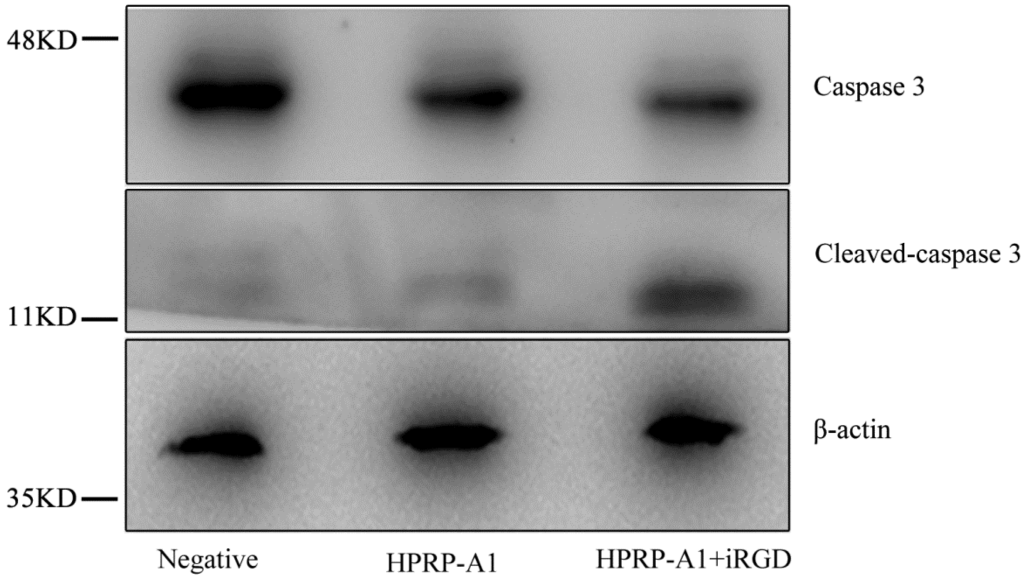

Figure S2. The representative western blotting bands of Caspase 3 and Cleaved-caspase 3 of A549 Cells treated with 4 μM HPRP-A1 with or without 64 μM iRGD for 1 h. The bands of Caspase 3 and Cleaved-caspase 3 were cropped from one gel, as shown with indication of molecular size.

Supplementary Video S1-S4

Video S1: Video of cellular uptake of 8 µM HPRP-A1 treatment. A549 cells were stained by Hoechst 33258. 8 µM of FITC-labeled HPRP-A1 were added after scanning the cells for two cycles, and the images were scanned for 10 min on the speed of 10 s per image. The fluorescence was measured using a laser scanning confocal microscope (LSCM). Blue color denotes nuclei, green color denotes FITC-modified peptide. The video was produced based on 600 images.

Video S2: Video of cellular uptake of 8 µM HPRP-A1 co-administration with 64 µM iRGD treatment. A549 cells were stained by Hoechst 33258. 8 µM FITC-labeled HPRP-A1 co-administration with 64 µM iRGD were added after scanning the cells for two cycles, and the images were scanned for 10 min on the speed of 10 s per image. The fluorescence was measured using a laser scanning confocal microscope (LSCM). Blue color denotes nuclei, green color denotes FITC-modified peptide. The video was produced based on 600 images.

Video S3: Video of cellular uptake of 16 µM HPRP-A1 treatment. A549 cells were stained by Hoechst 33258. 16 µM of FITC-labeled HPRP-A1 were added after scanning the cells for two cycles, and the images were scanned for 10 min on the speed of 10 s per image. The fluorescence was measured using a laser scanning confocal microscope (LSCM). Blue color denotes nuclei, green color denotes FITC-modified peptide. The video was produced based on 600 images.

Video S4: Video of cellular uptake of 16 µM HPRP-A1 co-administration with 64 µM iRGD treatment. A549 cells were stained by Hoechst 33258. 16 µM FITC-labeled HPRP-A1 co-administration with 64 µM iRGD were added after scanning the cells for two cycles, and the images were scanned for 10 min on the speed of 10 s per image. The fluorescence was measured using a laser scanning

confocal microscope (LSCM). Blue color denotes nuclei, green color denotes FITC-modified peptide. The video was produced based on 600 images.
